# Parasitic mussels induce upstream movement in their fish hosts: early evidence of extended phenotype

**DOI:** 10.1093/beheco/araf043

**Published:** 2025-05-04

**Authors:** Sebastian L Rock, P Anders Nilsson, Johan Watz, Olle Calles, Martin Österling

**Affiliations:** River Ecology and Management, Research Group RivEM, Department of Environmental and Life Sciences, Karlstad University, Universitetsgatan 2, Karlstad, 65188, Sweden; Lund University, Department of Biology - Aquatic Ecology, Ekologihuset, Kontaktvägen 10, Lund 223 62, Sweden; River Ecology and Management, Research Group RivEM, Department of Environmental and Life Sciences, Karlstad University, Universitetsgatan 2, Karlstad, 65188, Sweden; River Ecology and Management, Research Group RivEM, Department of Environmental and Life Sciences, Karlstad University, Universitetsgatan 2, Karlstad, 65188, Sweden; River Ecology and Management, Research Group RivEM, Department of Environmental and Life Sciences, Karlstad University, Universitetsgatan 2, Karlstad, 65188, Sweden

**Keywords:** behavior, conservation, ecology, host manipulation, *Margaritifera margaritifera*, *Salmo trutta*, Salmonidae, unionida

## Abstract

Parasites often have a large impact on their hosts and can alter host phenotype to increase their own fitness, a phenomenon known as *extended phenotype*. Studies demonstrating extended phenotype for non-trophically transmitted parasites are scarce. Unionid mussels have a parasitic life stage adapted to parasitize fish which can affect host behavior, habitat use and growth rates, raising the question if parasitic freshwater mussels can also manipulate their host fish to compensate for downstream dispersal and to reach habitats favorable for newly excysted juvenile mussels. Wild-caught, parasite-naïve juvenile brown trout (*Salmo trutta*) were PIT-tagged, and half of the individuals were infested with parasitic larvae from the freshwater pearl mussel (*Margaritifera margaritifera*), all individuals were then returned to their home stream. During the following year, trout were tracked to investigate movement and habitat use, and also periodically recaptured to measure growth and body condition factor. The infested trout showed significantly higher upstream movement than non-infested trout and were more often recaptured in stream sections with slow-moving shallow water, particularly during the parasite excystment period (270 d post infestation). These data suggest that the juvenile mussels were successfully transported an average of 170 m upstream from the host trout release points to stream sections favorable for adult mussels. Infested trout survived as well as the non-infested, but had a significantly lower specific growth rate than non-infested trout. These results indicate a first example of extended phenotype in unionid mussels and highlight the importance of understanding glochidia-induced changes to host fish behavioral ecology.

## Teaser

Parasites often manipulate host behavior to increase their reproductive success, but studies on this phenomenon typically focus on parasites which lead to the eventual death of their host, an effect that parasitic freshwater mussels generally do not have. Here, we show that parasitic freshwater mussel larvae manipulate host fish into swimming further upriver to habitats well suited for the next stage of their lifecycle.

## Introduction

Parasitized hosts are often observed as behaving differently from their unparasitized counterparts (Moore 2013). Many of these behavioral changes can be a consequence of the stress from parasitism or anti-parasite tactics by the infested host (Wisenden et al., 2019). The parasite itself may also induce behavioral changes in the host; when the induced effects on hosts are beneficial to the parasite, the parasite is considered to manipulate its host, showing a first step towards the evolution of an extended phenotype (Moore, 2002, 2013; Poulin 2007). For example, the protozoan *Toxoplasma gondii* alters its rodent host behavior to make the infected animal lose its fear of cats, the definitive host, and instead seek them out (Berdoy et al., 2000; Webster 2007). Certain parasitic nematodes will induce suicidal drowning in their cricket hosts, allowing the nematode to reach an aquatic habitat where it can complete its life cycle (Thomas et al., 2002; Biron et al., 2006). Given the diversity of possible effects parasites may have on their hosts, and the mechanisms involved in the manipulation, the study of these interactions can prove valuable in understanding the evolution of parasite-host biology, ecology and behavior (Poulin 2007).

Studies on extended phenotype have mostly been performed in regards to trophically transmitted parasites (Hughes 2013). In contrast, in cases where the parasite does not lead to host death, such as with parasitic freshwater bivalves (Order: Unionida), studies of extended phenotype are scarce, and completely lacking for unionids (Rock et al., 2022). Unionid mussels have a parasitic larval life stage, known as a glochidium, which affixes to the exterior surfaces of host fish, typically the gills, and remains encysted for a period of time before it metamorphoses and excysts, falling to the substrate as a free-living mussel. Most unionids live in flowing waters where males release sperm into the water column to reach downstream females for fertilization. Generally, females release larval glochidia that follow the water flow to encounter downstream host fish, though many species use sophisticated lure mechanisms to manipulate hosts into ingesting the glochidia directly (Barnhart et al., 2008; Strayer 2008; Rock 2024). Drifting glochidia can typically be found up to approximately 100 m downstream from the mother (Schwalb et al., 2010, 2012). For unionid mussels living in rivers, this reproductive strategy, in combination with the limited movement and dispersal capacities of adult mussels, should result in a net downstream movement of mussel distribution over subsequent generations, likely away from suitable lotic habitats. The mussel usage of their host fish as nutrient and energy source can reduce host growth, which may relate to differences in dispersal between infested and non-infested fish (Horký et al., 2014; Denic et al., 2015; Terui et al., 2017). If encysted glochidia could express an extended phenotype by inducing upstream movement in their host fish in time for the excystment, this would enhance the possibility of mussels remaining in suitable habitats. Unionids rely on host survival for the duration of the infestation period to complete a generational turnover as host death leads to the death of all encysted glochidia. Therefore, excessive harm to the host is not a beneficial adaptation for mussels with a glochidium life stage. It has even been reported that body condition in host salmonids can be improved from infestation of the freshwater pearl mussel *Margaritifera margaritifera,* which could indicate an extended phenotype (Ziuganov 2005; Marwaha et al. 2019).

The European freshwater pearl mussel (*M. margaritifera*), is highly host specific, targeting Atlantic salmon (*Salmo salar*) and brown trout (*S. trutta*), and remains encysted for approximately 9 to 10 mo, ie approximately 270 d post infestation (dpi—Young and Williams 1983; Skinner et al., 2003; Geist et al., 2005; Salonen et al., 2017; Salonen and Taskinen 2017; Marwaha et al., 2019; Wacker et al., 2019). The species is almost exclusively found in lotic habitats, is considered highly endangered, and many populations have become locally extinct (Lopes-Lima et al., 2017). Therefore, some populations of stationary (non-migratory) brown trout have not been in contact with freshwater pearl mussels for multiple generations (Jonsson and Jonsson 2011). Streams with such trout populations constitute ideal study systems to test for extended phenotypes in Unionids, as brown trout that are guaranteed to be parasite-naïve can be artificially infected with mussel glochidia, tagged, and then monitored for movement behavior, habitat use, body condition and growth in their natural stream for comparisons with non-infested trout of the same population. Furthermore, as adult freshwater mussels are well recognized ecosystem engineers (Zieritz et al., 2022), their absence from the study system helps control for any confounding impacts the adults may have, such as altering fish distributions, benthic fauna community composition and periphyton density (Spooner and Vaughn 2006; Hopper et al., 2019).

The present study was made possible by a reintroduction effort for freshwater pearl mussels in the Skärån stream in the Söderåsen National Park, southern Sweden, where the pearl mussel has been locally extinct for several hundred years whereas the resident brown trout population remains. Skärån trout were caught, PIT-tagged, half of them artificially infested with freshwater pearl mussel glochidia from a nearby population, and then returned to their home stretch: a common reintroduction strategy for unionids (Brian et al., 2021). The trout were regularly tracked and recaptured to monitor growth, body condition, movement and habitat use. If freshwater pearl mussel glochidia were to extend their phenotype via its host trout, we predicted that, despite likely lower growth and body condition, infested brown trout would move further upstream and reside in more suitable mussel habitats in time for glochidia to excyst compared with non-infested trout.

## Methods

### Host trout collection and maintenance

**Fig. 1.**
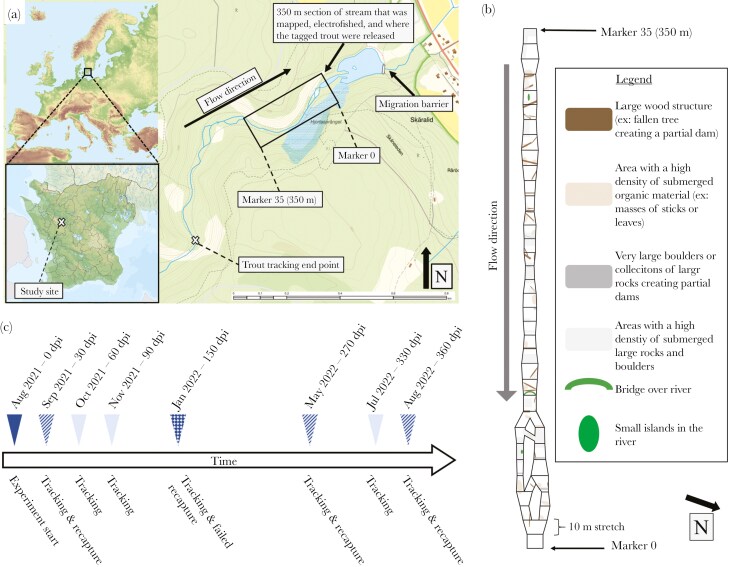
Experimental location in Söderåsen national park (a), the simplified map used to identify trout location within the stream, annotated with some major structural elements (b) and experiment timeline of tracking and recapture events at different days post infestation (dpi) (c). In (c): dark shade designates the start of the experiment, light shades designate tracking events, stripe fill indicate successful recapture events, square fill indicate unsuccessful recapture events.

Stationary brown trout were electrofished between August 5 and 9, 2021 (mean length ± SD = 14.82 ± 2.96 cm, Table 1), from a 350 m stretch of the Skärån stream in Söderåsen National Park (56°2’13.2”N, 13°14’51.2”E WGS84; Fig. 1a), part of the Rönne å river catchment. This stretch corresponded to the first 350 m section of the Skärån stream as it fed into a small lake, man-made from damming the stream. A nature-like fishway was constructed around the dam in 2018, but anadromous trout cannot reach it due to migration barriers further downstream. Caught trout were housed indoors in two 500 L aquaculture holding tanks, filled with local stream water. Each tank was filtered with two large canister filters (EHEIM classic 1500 XL 2260, Ehime, Germany), each fitted with one large return pump (EHEIM universal 2400). Tanks were kept at 16 °C with two chillers each (TK 2000, TECO, Italy), fitted with similar return pumps. All four return pipes were directionally placed above the water surface to ensure high water oxygenation and circular water flow in the tanks. Each holding tank was covered in netting to prevent the trout from jumping out of the water, with a further covering of polystyrene sheets that insulated the water temperature and provided cover for the trout. Several sections of large aquaponics filter material were floated in the water to provide some visual breaks in the tank and further discourage jumping. Trout were fed to satiation every second day with a mixture of frozen chironomid larvae and fly maggots. Water changes of approximately 50% were performed every day, and uneaten food was removed during these changes. Ethical approval for this study was obtained by Karlstad University (001673 - Göteborgs djurförsöketiska nämnd).

**Table 1.** Number of trout (N), average trout length (cm ± SD) and average trout weight (g ± SD) of initial experimental trout cohort and subsequent resampled groups at different days post infestation (dpi) via the tracking procedure (T), or the electrofishing procedure (E). Table rows additionally separate these data by: Total trout cohort (Total), young-of-the-year trout (0+), one summer one trout (1+), two or more summer old trout (2+), each of which are additionally separated by infestation status.

Trout group	0 dpi			30 dpi				60 dpi	90 dpi	150 dpi	270 dpi				330 dpi	360 dpi			
	Starting cohort			T	E			T	T	T	T	E			T	T	E		
	N	cm ± SD	g ± SD	N	N	cm ± SD	g ± SD	N	N	N	N	N	cm ± SD	g ± SD	N	N	N	cm ± SD	g ± SD
Total non-infested	124	14.1 ± 0.9	31.0 ± 26.3	68	49	16.2 ± 2.0	38.5 ± 19.9	25	17	18	26	16	15.8 ± 3.4	20.6 ± 12.1	15	4	2	15.6 ± 3.3	14.1 ± 14.5
Total infested	151	14.8 ± 3.1	32.7 ± 19.1	83	57	15.3 ± 1.6	32.6 ± 10.2	32	17	19	35	19	15.9 ± 1.9	28.5 ± 10.3	17	6	6	18.2 ± 0.5	34.4 ± 6
0+ non-infested	22	7.1 ± 0.5	3.4 ± 0.9	5	-	-	-	1	4	4	7	5	10.9 ± 0.4	3.8 ± 0.6	6	1	1	13.3	3.8
0+ infested	14	7.4 ± 0.6	3.9 ± 0.7	4	1	8.8	3.9	2	1	3	2	2	11.3 ± 1.1	4.2 ± 1.0	2	-	-	-	-
1+ non-infested	96	15.1 ± 1.4	32.0 ± 9.0	59	45	15.7 ± 1.3	33.4 ± 8.6	22	13	14	19	11	18.0 ± 0.8	59.1 ± 5.7	9	3	1	18.0	53.8
1+ infested	130	15.1 ± 1.4	32.3 ± 8.2	77	55	15.4 ± 1.2	32.3 ± 7.7	28	16	15	33	17	16.5 ± 1.0	48.4 ± 8.9	15	6	6	18.2 ± 0.5	56.08 ± 6.4
2+ non-infested	6	22.7 ± 3.1	116.1 ± 58.4	4	4	21.3 ± 1.4	96.1 ± 20.8	2	-	-	-	-	-	-	-	-	-	-	-
2+ infested	7	22.3 ± 1.9	97.8 ± 28.0	2	1	21.0	76.6	2	-	1	-	-	-	-	-	-	-	-	-

### Mussel collection and maintenance

Gravid mussels were collected from the geographically closest population of freshwater pearl mussel in the nearby stream Klingstorpabäcken, also part of the Rönne å catchment area, on August 11, 2021. This location was chosen following the IUCN guidelines for reintroductions (IUCN/SSC 2013). Gravidity was determined by carefully opening the mussels with specialized tongs and visually inspecting the gill flaps for marsupial pouches (Österling et al., 2008). Five mussels with large marsupial pouches and well-developed glochidia were brought to the holding facility where they were kept in two well-aerated 10 L buckets of water, placed inside one of the trout holding tanks to calibrate temperature. The mussels in one of the buckets released glochidia on August 12, 2021, and were used in the infestation procedure (see “Trout handling”). After visual inspection of successful infestation on the trout the day after infestation, the mussels were relocated back to their home stream stretches.

### Trout handling

On August 10 to 11, 2021, all trout were tagged with 12 mm radio-frequency identification (RFID) passive integrated transponder (PIT) tags (12 mm x 2.15 mm FDX-B XL, Oregon RFID, Oregon, USA). Trout were sedated with benzocaine (100 mg/L, Sigma-Aldrich, USA), after which they were held belly up on a damp cloth and had a PIT-tag inserted into their coelomic cavity with the aid of a specialized syringe, as per Cook et al. (2014). After tagging, body mass (± 0.1 g) and total length from snout to the tip of the caudal fin (± 1 mm) was recorded for each individual. Trout were then placed in an aerated recovery tank before being returned to the holding tank.

On August 12, 2021, 161 trout were infested with glochidia, leaving a remaining 138 trout as non-infested controls. To randomly select trout for either treatment or control, all individuals were put into one tank from which they were netted out in groups (2 to 6 fish per net); nets were alternately allocated to the control or treatment group. Glochidium density and viability in the release bucket were first determined by counting the number of glochidia in a 0.5 ml drop under a stereo microscope and then adding a few grains of table salt (NaCl) to initiate a characteristic “snapping” stress reaction; this differentiated dead and living glochidia. The entire glochidia suspension (including non-snapping glochidia) was then diluted to 1500 living glochidia per liter (gl/L) and divided between five 60 L plastic tanks with vigorous aeration to ensure the glochidia stayed suspended. Approximately 30 trout were placed in these infestation chambers for 1 h before they were returned to their holding tanks. Control trout were subjected to a sham treatment by placing approximately 30 trout in five vigorously aerated 60 L plastic tanks free of glochidia for 1 h.

The day after the infestation procedure had been carried out, infestation success was examined on all test trout. Trout were lightly anesthetised with benzocaine and placed under a stereomicroscope; the left operculum was then gently lifted to allow for visual inspection of the infestation (Österling 2011). The infestation assessment demonstrated that all glochidia-treated trout were successfully infested with glochidia. Infestation success was too high to count without excessive harm to the host trout, but was estimated to be at minimum 1000 glochidia per individual. This level of infestation is very high, however not unnaturally so and has been previously demonstrated to not significantly increase host mortality (Chowdhury et al., 2019). Non-infested control trout were subjected to a sham treatment: following a light anesthesia with benzocaine, trout were placed under a stereomicroscope and the left operculum was gently lifted, a process which lasted roughly 10 s. Trout were randomly released in the same 350 m stretch of stream from where they were caught. Release positions were chosen haphazardly by gently pouring fish into a smaller net, identifying the individual and noting the stream section before releasing the trout. Care was taken to not release the largest trout in the shallowest stream sections.

### Stream mapping

A section of the study stream (indicated in Fig. 1a, expanded in Fig. 1b) was mapped in August 2021 with a protocol inspired by Wengström et al. (2022) and a national Swedish protocol (Länsstyrelsen Jönköping 2017). In short, the 350-m-long stretch was divided into 35 sections (length = 10 m), within which several key habitat features were categorized: dominating substrate type, secondary substrate type, dominating flow condition, average and maximum depth, stream width and longitudinal distance from the downstream lake. These parameters were recorded every 5 m at three points in transects across the stream and averaged to categorize the 10-m-long sections. Certain stretches of the stream split in two before re-joining; each fork was given a unique section ID and mapped independently.

### Trout monitoring

Over the course of 1 yr, seven tracking events were performed (Fig. 1c). The stream was waded in an upstream direction with a portable PIT-tag scanning antenna (Oregon RFID). The stream stretch upstream of the 350 m release area was also scanned with the portable antenna (~1.5 km upstream) but not mapped. The area downstream from the release area was neither scanned nor mapped, the muddy substrate and deep water in this section made tracking impractical and dangerous, particularly during winter at high water levels and flow (Watz et al., 2016). Moreover, these habitats are generally unsuitable for the sizes of trout used in our study (Jonsson 1989). When found, the location of a PIT-tag was recorded on a paper map (Fig. 1b), when found beyond the mapped section, the location was recorded on a larger scale map (Fig. 1a) later digitized with ImageJ. The stream Skärån is located in a deep, narrow valley that made GPS positioning less reliable than a paper map. The installation of permanent data loggers and PIT-antenna was not permitted given the protected status of the stream and Söderåsen National Park, and impractical given the high tourist density.

Electrofishing was performed immediately after each of four tracking surveys, though only three were successful due to unsuitable river conditions in winter (Fig. 1c). These recapture procedures were only performed within the mapped area of stream, with the exception of the final electrofishing event which specifically targeted a pool roughly 400 m upstream of the mapped section where several trout were recorded with the tracking procedure. Every survey used two-pass electrofishing, and took 2 d to complete. Upon capture, trout were held in aerated buckets, separated by 5 m increments as to return each individual to the area of capture. When a tagged trout was found, body mass, length and infestation status were recorded as before, following a light anesthesia with benzocaine. By 270 dpi, during the start of the period of mussel excystment, 12 previously infested trout had between 0 and 10 visible glochidia, three had over 10 glochidia, five had over 100, and two had over 1000.

### Data analysis

**Fig. 2.**
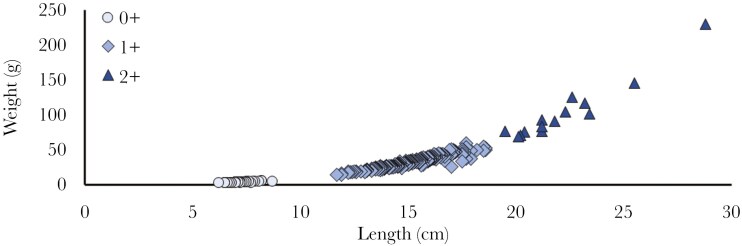
Relationship between length (cm) and weight (g) of all trout caught from Skärån stream. Different marker types correspond to different size classes: Young-of-the-year (0+; circle), one summer old (1+; diamond), two or more summer old (2+; triangle).

Salmonids have relatively small home ranges, but can move great distances between seasons (Hesthagen 1990; Höjesjö et al., 2007; Stakėnas et al., 2013; Radinger and Wolter 2014). Thus a trout was considered dead when it was not recaptured and was recorded in the same section or two adjacent sections (only when the tag moved downstream) for three or more tracking events in a row. The first of these recordings were kept in the dataset, all future records were removed (including 8 non-infested and 16 infested trout total at 360 dpi). Trout which did not change sections between the July and August tracking surveys (two last months of the study), and were not recaptured in August, were removed from the August data. Only one-summer-old individuals (1+), defined by length at first capture (size range determined from visual inspection of size distribution: 11.7 to 19.5 cm; Fig. 2) were used in the data analysis. This decision was made because of the low starting number and low recapture rates of the other size classes (Table 1). Analyses including all size classes are reported in Supplement 1.

Effects of infestation on survival was tested using separate Fisher’s exact tests for each tracking event, using detection rates as a proxy measure for survival. If the detection rates of infested and non-infested trout were consistent through time, we assumed equal survival rates. Specific growth rate (SGR; proportional change in mass over time) was calculated as described by Crane et al., (2020) and was used to assess trout growth; change in trout body mass was always calculated in relation to mass at initial release. Fulton’s condition factor (K) was used to assess the change in trout body condition over time, also calculated in relation to mass and length at initial release. Trout total length was used to track changes in overall size over time. Distance moved (DM) was measured in 10 m increments relative to the initial release location and was calculated by subtracting the release section number from that of each re-capture section. Positive values correspond to upstream movement and negative values downstream movement; for example, if a trout was released in section 10 but recovered in section 8 at 30 dpi, it was considered to have moved −20 m at 30 dpi. If it was then recovered in section 25 at 60 dpi it was considered to have moved + 150 m at 60 dpi. Measuring distance moved from the release location was used as opposed to calculating the between-tracking distance moved as individual trout were not consistently recovered every tracking event. For example, if a trout was not recovered at 30 dpi, but was recovered at 60 dpi, no between-tracking distance moved could be calculated for either 30 dpi or 60 dpi for that individual.

For SGR, K, total length, mass and distance moved, linear mixed models (LMM) with a heterogeneous compound symmetry error distribution were fitted to the data using infestation (yes/no), dpi, and the interaction between infestation and dpi as predictors. The characteristics of habitat type were first simplified with a principal component analysis with varimax rotation. Three separate LMMs with homogeneous compound symmetry error distribution were then fitted to the regression factor scores (RFS) of the first three rotated components (RPC), again using infestation, dpi, the interaction between infestation and dpi. This allowed us to more easily compare the variation between infested and non-infested trout habitat preference, as the characteristics of the habitat types measured here are not independent of each other (ex: finer sediments accumulate in areas of slower flow). Post-hoc pairwise comparisons for all LMMs were performed using the least significant difference. Analyses including starting length as a covariate are reported in Supplement 1. All statistical analysis was performed with IBM SPSS Statistics V28.

## Results

### Trout biometrics

**Fig. 3.**
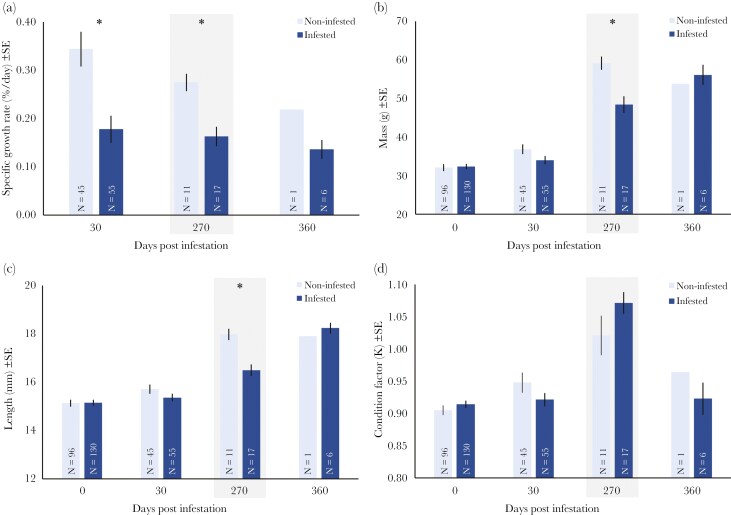
Specific growth rate (SGR; a), mass (b), total length (c) and Fulton’s condition factor (K; d) of infested and non-infested one-summer old (1+) *S. trutta* over time. Asterisk indicates significant differences between treatments groups at p < 0.05, shaded section indicates excystment period, sample sizes indicated within each bar.

Infested trout had significantly lower SGR than the non-infested trout (Table 2, Fig. 3a), gaining half as much mass during the first 30 dpi (mean SGR ± SE = 0.18 ± 0.03% vs 0.34 ± 0.04% per day; respectively). This difference in SGR remained relatively consistent for the duration of the investigation period (Table 3, Fig. 3a). The significantly lower SGR of infested trout did not result in a significant difference in overall trout mass (Table 2), although there was a significant difference in trout mass at 270 dpi (Table 3, Fig. 3b). Despite similar overall mass, infested trout were shorter than non-infested trout (Table 2), with a significant difference at 270 dpi (Table 3, Fig. 3c). There was no difference in body condition factor K between the two trout groups (Table 2, Fig. 3d). There was no significant difference in detection rates between infested and non-infested trout for all tracking events (separate Fisher’s exact tests), indicating that infestation did not influence survival over the investigation period (Table 3).

**Table 2.** Degrees of freedom (DF), F-statistic and significance level of factors used in linear mixed models on specific growth rate (SGR), mass, total length, condition factor (K), distance moved (DM) and regression factors scores (RFS) of habitat use. Significant factors are shown in bold.

Test variable	Factor	DF	F	P
SGR	**infestation**	**1, 34.6**	**20.827**	**<0.001**
	**dpi**	**2, 18.3**	**5.154**	**0.017**
	interaction	2, 18.3	1.122	0.347
Mass	infestation	1, 10.4	0.595	0.458
	**dpi**	**3, 8.3**	**9.307**	**0.005**
	interaction	3, 8.3	2.126	0.172
Total length	**infestation**	**1, 43.9**	**9.458**	**0.004**
	**dpi**	**3, 21.4**	**138.885**	**<0.001**
	**interaction**	**3, 21.4**	**11.954**	**<0.001**
K	infestation	1, 4.8	0.031	0.868
	**dpi**	**3, 3.9**	**15.199**	**0.013**
	interaction	3, 3.9	1.431	0.360
DM	**infestation**	**1, 231.0**	**15.111**	**<0.001**
	**dpi**	**6, 249.4**	**7.877**	**<0.001**
	**interaction**	**6, 249.4**	**2.313**	**0.034**
RFS1	**infestation**	**1, 214.8**	**4.666**	**0.032**
	dpi	6, 187.3	1.815	0.098
	interaction	6, 187.3	0.901	0.495
RFS2	infestation	1, 211.5	1.316	0.253
	**dpi**	**6, 192.4**	**3.637**	**0.002**
	interaction	6, 192.4	1.147	0.337
RFS3	infestation	1, 226.0	0.936	0.334
	dpi	2, 172.8	0.948	0.462
	interaction	2, 172.8	1.051	0.394

**Table 3.** Results of Fisher’s exact test on trout detection rates and denominator degrees of freedom (DF), F-statistic and significance level of pairwise comparisons on specific growth rate (SGR), mass, total length, condition factor (K), distance moved and regression factors scores (RFS) of habitat use between infested and non-infested *S. trutta* over time. Significant comparisons are shown in bold.

dpi	Detection rates	SGR			Mass			Total length			K			Distance moved			RSF1			RSF2			RSF3		
	Exact sig. (1- sided)	DF	F	P	DF	F	P	DF	F	P	DF	F	P	DF	F	P	DF	F	P	DF	F	P	DF	F	P
0	-	-	-	-	224.6	0.064	0.800	214.8	0.009	0.924	224.1	0.998	0.319	-	-	-	-	-	-	-	-	-	-	-	-
30	0.421	**89.6**	**12.579**	**<0.001**	81.81	0.016	0.899	138.2	0.200	0.656	135.1	2.129	0.147	297.3	1.499	0.222	238.9	0.247	0.620	241.4	0.008	0.927	229.2	0.003	0.954
60	0.539	-	-	-	-	-	-	-	-	-	-	-	-	**309.1**	**13.686**	**<0.001**	240.5	2.003	0.158	242.4	3.785	0.053	235.5	0.117	0.733
90	0.468	-	-	-	-	-	-	-	-	-	-	-	-	**290.9**	**5.843**	**0.016**	216.6	0.030	0.863	220.8	0.019	0.890	205.9	0.000	0.989
150	0.315	-	-	-	-	-	-	-	-	-	-	-	-	298.2	1.291	0.257	216.6	2.271	0.133	220.4	0.724	0.396	207.1	0.195	0.660
270	0.204	**28.7**	**9.861**	**<0.001**	**5157.4**	**5.771**	**0.016**	**55.998**	**23.942**	**<0.001**	16.0	0.415	0.528	**311.3**	**11.713**	**<0.001**	245.5	0.120	0.730	**246.8**	**4.003**	**0.047**	242.2	4.107	0.044
330	0.384	-	-	-	-	-	-	-	-	-	-	-	-	274.4	0.027	0.871	211.8	0.661	0.417	216.1	0.017	0.895	201.8	0.049	0.826
360	0.581	6.1	2.289	0.181	3.4	0.777	0.435	8.5	3.137	0.112	2.5	0.020	0.899	301.3	2.539	0.112	213.9	3.194	0.075	218.1	0.019	0.891	201.1	1.743	0.188

### Trout movement

**Fig 4.**
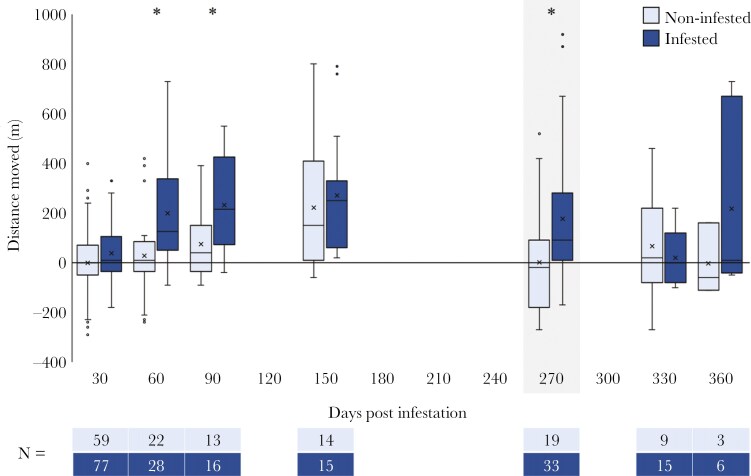
Box & whisker plot of distance moved by infested and non-infested one-summer old (1+) *S. trutta* over 1 yr. Asterisk indicates significant differences between treatments groups at p < 0.05, shaded section indicates excystment.

Infested trout moved significantly farther and earlier than the non-infested trout (Table 2; Fig. 4). At 30 dpi, infested trout had moved upstream by 38 ± 6 m (mean ± SE), whereas the non-infested trout had moved downstream −1 ± 17 m. By 60 dpi, infested trout had moved 198 ± 19 m upstream whereas the non-infested trout had moved only 28 ± 18 m upstream. At 90 dpi the two trout groups maintained a large difference in dispersal (157 m) despite both moving slightly further upstream (DM_infested_ = 231 ± 36 m; DM_non-infested_ = 73 ± 40 m). At 150 dpi, both groups achieved their furthest upstream movement, reaching roughly the same distance (DM_infested_ = 271 ± 64 m; DM_non-infested_ = 221 ± 68 m), explaining the significant infestation × dpi interaction term (Table 2). At 270 dpi, the non-infested trout had more or less returned to their origin (2 ± 52 m), while the infested trout were still 178 ± 46 m upstream from their release location. This difference rapidly shrank when the infested trout moved roughly 150 m downstream by 330 dpi (ie after glochidia excystment). By 360 dpi infested trout apparently moved more than 150 m back upriver. The low sample size from this tracking event makes this result less trustworthy as it is primarily driven by two individuals not recorded at 330 dpi which were recorded very far upstream at 360 dpi. Significant pairwise comparisons were present at 60, 90 and 270 dpi (Table 3).

### Variation in stream habitat

The principal component analysis on habitat metrics indicated a strong correlation between the flow condition and the primary substrate (r = 0.619) and a very strong correlation between average depth and maximum depth (r = 0.914). The rotated component matrix further indicated that these two pairs of correlated metrics positively contributed most to RPC1 (r_flow condition_ = 0.867, r_primary substrate_ = 0.799) and RPC2 (r_average depth_ = 0.956, r_mean depth_ = 0.938), collectively describing 68.16% of total variance in habitat characteristics. RPC3 described an additional 14.71% of habitat variance and was primarily driven by stream width (r = 0.925), but did not strongly correlate with any other metric. These three rotated components collectively described 82.87% of total variance of habitat in the stream. RPC1 has a straightforward interpretation; sections with high RFS1 have fast flowing water and are dominated by larger substrate categories, whereas sections with low RFS1 have slow flowing water and small substrates. Both RPC2 and RPC3 describe habitat metrics measured on a continuous scale. High RFS2 scores correspond to deep water, whereas high RFS3 values correspond to wide stream sections. A difference in RFS2 of 1 is roughly equivalent to a 10 cm difference in water depth, whereas a difference in RFS3 of 1 is roughly equivalent to a 4 m difference in section width. The addition of other habitat descriptors did not produce significant increases in captured variance or align well with further rotated components.

### Trout habitat use

**Fig 5.**
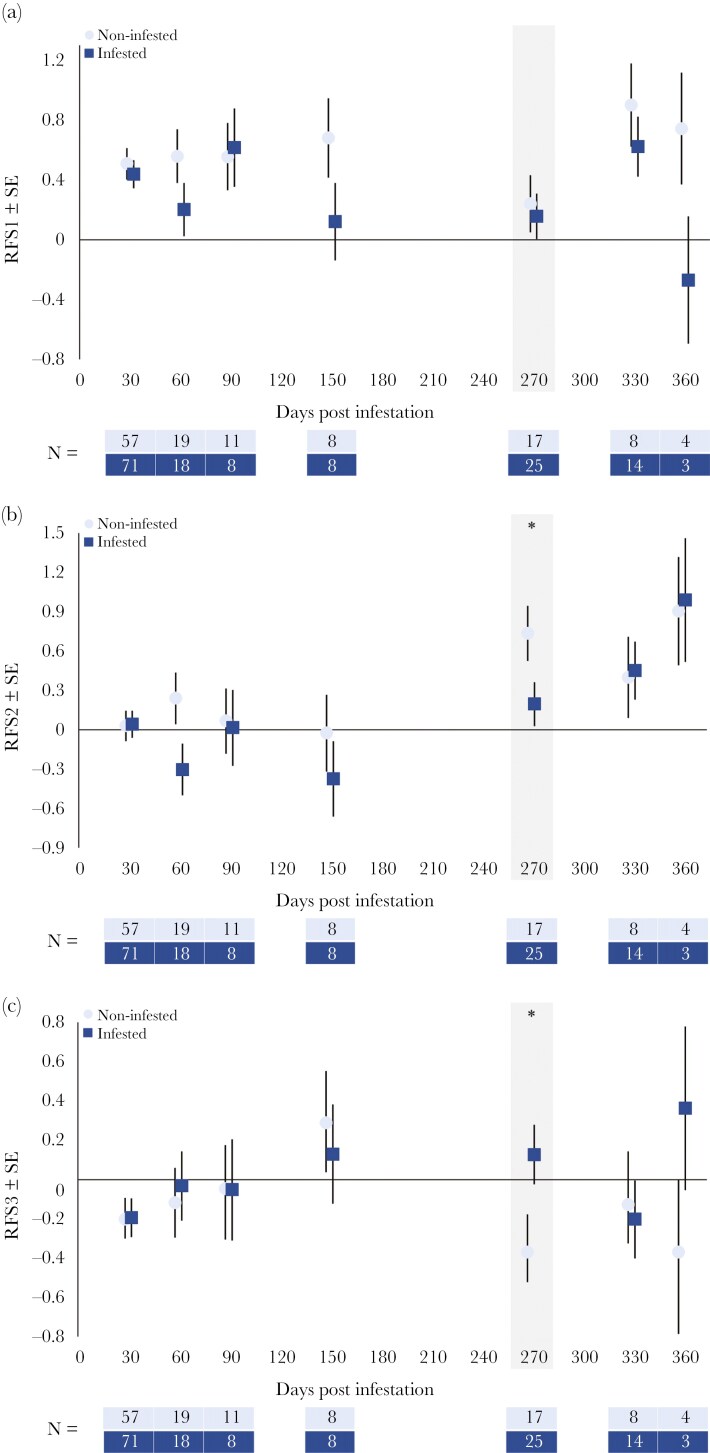
Average regression factor score (RFS) of habitats used by infested and non-infested one-summer old (1+) *S. trutta* over time for rotated components (RC) 1, 2 and 3. a) RPC1, flow condition and primary Substrate. b) RPC2, water depth. c) RPC3, section width. Asterisk indicates significant differences between treatments groups at p < 0.05, shaded section indicates excystment.

Non-infested trout were more often found in habitats with significantly larger substrate categories and faster flow conditions than infested trout (RFS1; Table 2, Fig. 5a). However, at 270 dpi, when the juvenile mussels detach from their hosts, no significant difference was observed between infestation groups.

There was no significant difference between non-infested and infested trout along RFS2 (water depth), though a significant temporal trend was apparent (Table 2). Generally, trout were found in sections with higher RFS2 as time passed, particularly from 270 dpi onwards (Fig. 5b). A significant pairwise difference between infestation groups was apparent at 270 dpi (Table 3), where infested trout were recorded more often in habitats with lower RFS2 (shallower water).

The LMM on RFS3 (river width) showed that neither infestation nor dpi had significant effects in predicting trout habitat use (Table 2, Fig. 5c). A significant difference between infestation groups was apparent at 270 dpi (Table 3), with infested trout more often found in habitats with higher RFS3 (wider stream sections).

## Discussion

The results presented here show that a non-trophically transmitted parasite, which does not benefit from host mortality, may display an extended phenotype on their host. Infested trout displace further upstream earlier in the year than non-infested individuals, with a noteworthy difference around the time of mussel excystment, which coincides with significant differences in habitat use. This upstream movement of juvenile mussels via host fish likely conveys a fitness benefit to the mussels by counteracting the net downstream displacement inherent to their life cycle, and transport of the juveniles to habitats suitable for growing into adult mussels (Jung et al., 2013). Heavy infestation of glochidia may increase mortality in hosts (Cunjak and McGalddery 1991). In our study, infested trout generally gained less size compared to non-infested individuals, without apparent effects on trout body condition or survival. Below, we propose an expression of extended phenotype whereby the freshwater pearl mussel decreases host growth to take advantage of the size-dependent aggression in their salmonid hosts (Fausch and White 1986; Kennedy and Strange 1986).

Infestation has been previously demonstrated to decrease both dominance and drift-feeding ability in infested trout (Österling et al., 2014; Filipsson et al., 2016). Here, we show that infested trout use habitats with lower water velocities, in line with previously published work on swimming condition and activity levels (Taeubert and Geist 2013; Filipsson et al., 2016). The reduced swimming activity, combined with previously reported increases to metabolic rate (Thomas et al., 2014; Filipsson et al., 2017) and decreases in drift-feeding ability (Österling et al., 2014) likely explain the decreased specific growth rate seen here, particularly in the first 30 dpi when the negative effect from infestation is highest (Rock et al., 2022), ultimately reducing the total length of our infested trout. Infested trout gain almost no mass or length in the first 30 dpi, which was likely caused by high glochidia loads. Similar decreases in growth with no impact to survival have been demonstrated in laboratory conditions (Chowdhury et al., 2019).

Salmonids have well documented dominance interactions, particularly across sizes, with smaller individuals generally being less dominant than larger ones, often forcing them to relocate (Fausch and White 1986; Kennedy and Strange 1986; Gunckel et al., 2002; Nilsson et al., 2004; Näslund and Johnsson 2016). Larger dominant salmonids generally place themselves upstream of smaller less dominant individuals to secure more favorable foraging positions (Gunckel et al., 2002; Nilsson et al., 2004), with downriver moving trout often having comparable condition factors as stationary ones, but showing signs of starvation under molecular analysis (Elliott 1993). Moreover, migratory behavior is partly a phenotypically plastic response influenced by environmental conditions such as food availability, with lower growth rates corresponding to higher downstream migration (Olsson et al., 2006). Infested trout typically demonstrate subordinate behavior and lower feeding rates (Österling et al., 2014; Filipsson et al., 2016), though some evidence proposes that infested trout may initiate and win more aggressive acts against non-infested conspecifics (Rinnevalli 2018).

The greater upstream movement of infested host fish, particularly at the time of excystment, is the clearest indication of an extended phenotype expressed by glochidia presented here, as our trout appear to behave in a manner opposite to what should be observed, ultimately benefitting the encysted mussels. Had our trout behaved in a manner as suggested by Elliott (1993), we should have observed higher downstream movement by infested individuals. Salmonids infested with *Margaritifera* sp. have been generally shown to disperse less than their non-infested counterparts, but when relocating, relocating further in both directions (Teuri et al., 2017; Horký et al., 2019, Rock et al., 2022). Our site selection and sampling method suffered from some logistical limitations and may have biased our results. By placing the starting point of our release and tracking stretch immediately upriver of habitat unsuitable for juvenile trout, the only direction infested trout could move to avoid non-infested counterparts would be upstream, thus potentially confounding generally higher dispersal with higher upstream dispersal. Higher dispersal would still be a fitness advantage to the encysted glochidia as by chance some would relocate further (Teuri et al., 2014; Terui and Miyazaki 2015). Regardless, our data demonstrates that our, assumed, less dominant infested trout moved further upstream to stream areas more suitable for adult mussels than others than our, assumed, more dominant non-infested trout.

As the reproductive strategy of the freshwater pearl mussel transports sperm and glochidia downstream, and the adult life stage has limited mobility, hitchhiking to low-flow stream sections, upstream of where the hosts were initially infested, allows young mussels to settle in habitats suited for later development into adults (Jung et al., 2013). Moreover, the average distance moved by infested trout by the time of excystment (170 m) is further than the reported downstream dispersal of glochidia in the water column (< 100 m; Schwalb et al., 2010, 2012), indicating that the upstream movement by infested trout could offset the downstream drift of the larval glochidia. Infested trout move further upstream than their non-infested counterparts at all stages of the encystment period, with the exception of 150 dpi. We argue that this is a result of our sampling technique where trout likely remained stationary and buried into the substrate during winter where they were difficult to detect (Watz et al., 2016; 2019). This was supported by our observation of higher sample sizes at 270 dpi when the water temperatures were higher compared to 90 and 150 dpi.

The habitat use of infested and non-infested trout during this study showed significant all-year differences in use of flow condition, and significant differences in depth and width of habitat used at the time of excystment. We suggest two non-mutually exclusive hypotheses that would be favorable for mussel fitness. Firstly, slow flowing sections allow the recently excysted juvenile mussels to settle to the bottom and not be flushed back downriver. Moreover, the habitats used by the infested trout at 270 dpi are comparable to the habitats preferred by adult pearl mussels (Jung et al., 2013). Secondly, one can reasonably speculate that slow-flowing, wide and shallow sections may be warmer than fast-flowing, deep and narrow sections as a function of light penetration, though exact temperature data were not collected here. This is of noteworthy importance as juvenile pearl mussels require a thermal cue to excyst (Scheder et al., 2014; Eybe et al., 2015). Previous studies have documented that parasitized fish prefer warm waters; a phenomenon referred to as *behavioral fever*, common in many other instances of disease in ectothermic animals (MacNab and Barber 2012; Mohammed et al., 2016; Rakus et al., 2017; Boltana et al., 2018), although the literature in relation to glochidiosis is still unclear (Horký et al., 2019). Additionally, juvenile mussel excystment has been demonstrated to be more damaging to the gill structure than the initial encystment of glochidia (Rock and Townsend 2025), likely causing a significantly larger shift in host behaviour around 270 dpi than at earlier stages. While we cannot provide evidence for a definitive mechanism of manipulation, we suggest that *M. margaritifera* can benefit from the natural response of fish to parasitism (preference for calmer, warmer water) to be transported to habitats with low flow and high temperature, ultimately allowing the juvenile mussels to settle in high-quality habitat after receiving a temperature cue to excyst. There is no practical way to distinguish between an advantageous product of selection or an advantageous byproduct of parasitism, regardless, the effect remains (Poulin 2010).

It has been suggested that Unionid mussels do not gain a fitness advantage from inducing significant negative impacts on their host fishes, as host mortality leads to the death of all encysted glochidia (Rock et al., 2022). This effect should be more evident in longer-infesting mussels, such as the freshwater pearl mussel, as the likelihood of host mortality increases over time (Rock et al., 2022). Evidence of increased host salmonid survival and condition has been previously demonstrated following pearl mussel glochidiosis (Ziuganov 2005; Marwaha et al., 2019). The combination of equal survival and lack of differences in condition factor between infested and non-infested trout that this study presents is commonly reported in other studies on glochidiosis in salmonids, and may indicate a stable-state solution to the arms race between parasite and host (Rock et al., 2022). The trout used here have been isolated from the selection pressure of freshwater pearl mussel for centuries, and have likely lost any adaptation to the effects of glochidiosis through genetic drift. Repeating a similar study on a population of trout adapted to glochidiosis may not result in as strong of a response.

Our results propose that a non-trophically transmitted parasite can express an extended phenotype by reducing host growth to take advantage of inter-individual dominance hierarchies. These results are of further specific value given that research on the ecology and parasitology of unionid mussels are both limited and in demand as conservation actions for freshwater mussels gain widespread international interest. This study was made possible by one such mussel reintroduction effort, which provided an ideal testing ground to investigate the extended phenotype. As the freshwater pearl mussel has been extinct in the Skärån stream for centuries, we were able to perform an in-situ study on parasite-naïve hosts with likely no adaptive defence mechanism against the parasite, in the absence of the ecosystem effects of the adult parasitic mussels. With that, further research on different age classes of fish, the effect of glochidia retention and re-infestation and the implication for future adaptive changes provide a fertile area for future studies.

## Supplementary Material

araf043_suppl_Supplementary_Materials_1

## Data Availability

Analyses reported in this article can be reproduced using the data provided by Rock et al. (2025).
