# Supplementary material for: Parasitic mussels induce upstream movement in their fish hosts: early evidence of extended phenotype
Source: Behav Ecol. 2025 May 4;36(4):araf043. doi: 10.1093/beheco/araf043 (PMC12228058; doi:10.1093/beheco/araf043)
Supplement: araf043_suppl_Supplementary_Materials_1 [file araf043_suppl_supplementary_materials_1.docx]

**Supplement 1**

All models presented in the main text (specific growth rate (SGR), mass, total length, condition factor (K), distance moved (DM) and regression factors scores (RFS) of habitat use) are re-presented here including all age classes of trout in the dataset and including starting length as a covariate in the model. The model for DM additionally includes trout release position as a covariate.

**Table 1:** Degrees of freedom (DF), F-statistic and significance level of factors used in linear mixed models on specific growth rate (SGR), mass, total length, condition factor (K), distance moved (DM) and regression factors scores (RFS) of habitat use. Significant factors are shown in bold.

| Test variable | Factor | DF | F | P |
| --- | --- | --- | --- | --- |
| SGR | **infestation** | **1, 23.9** | **22.499** | **<0.001** |
|  | **dpi** | **2, 16.9** | **4.544** | **0.026** |
|  | interaction | 2, 18.0 | 1.080 | 0.361 |
|  | **length** | **1, 41.8** | **167.855** | **<0.001** |
| Mass | **infestation** | **1, 273.2** | **12.157** | **<0.001** |
|  | **dpi** | **3, 78.2** | **133.187** | **<0.001** |
|  | **interaction** | **3, 78.2** | **6.674** | **<0.001** |
|  | **length** | **1, 248.7** | **1015.050** | **<0.001** |
| Total length | **infestation** | **1, 37509.7** | **92.749** | **<0.001** |
|  | **dpi** | **3, 6582.1** | **353.6** | **<0.001** |
|  | **interaction** | **3, 6544.3** | **31.161** | **<0.001** |
|  | **length** | **1, 269746.4** | **27259163983.790** | **<0.001** |
| K | infestation | 11.3 | 0.573 | 0.465 |
|  | **dpi** | **3, 8.5** | **25.302** | **<0.001** |
|  | interaction | 3, 8.6 | 1.273 | 0.344 |
|  | length | 1, 270.0 | 1.570 | 0.211 |
| DM | **infestation** | **1, 56.3** | **5.365** | **0.024** |
|  | **dpi** | **6, 51.1** | **6.372** | **<0.001** |
|  | interaction | 6, 51.0 | 1.652 | 0.152 |
|  | length | 1, 181.5 | 0.160 | 0.690 |
| RFS1 | infestation | 1, 50.7 | 2.878 | 0.096 |
|  | **dpi** | **6, 41.7** | **2.368** | **0.046** |
|  | interaction | 6, 42.0 | 1.129 | 0.362 |
|  | length | 1, 115.9 | 0.145 | 0.704 |
| RFS2 | infestation | 1, 289.0 | 0.112 | 0.738 |
|  | dpi | 6, 64.3 | 2.161 | 0.058 |
|  | interaction | 6, 73.8 | 1.168 | 0.333 |
|  | **length** | **1, 104.9** | **10.965** | **0.001** |
| RFS3 | infestation | 1, 64.9 | 0.005 | 0.943 |
|  | dpi | 6, 25.8 | 1.138 | 0.369 |
|  | interaction | 6, 26.2 | 1.452 | 2.33 |
|  | **length** | **1, 152.9** | **4.341** | **0.039** |

**Table 2:** Denominator degrees of freedom (DF), F-statistic and significance level of pairwise comparisons on specific growth rate (SGR), mass, total length, condition factor (K), distance moved and regression factors scores (RFS) of habitat use between infested and non-infested *S. trutta* over time. Significant comparisons are shown in bold.

| RFS3 | P | - | .770 | .993 | .747 | .858 | .108 | .096 | .458 |
| --- | --- | --- | --- | --- | --- | --- | --- | --- | --- |
|  | F | - | .085 | .000 | .107 | .033 | 2.655 | 2.914 | .607 |
|  | DF | - | 144.389 | 48.621 | 22.700 | 23.404 | 66.228 | 39.643 | 7.979 |
| RFS2 | P | - | .929 | **.029** | .839 | .484 | .323 | .777 | .457 |
|  | F | - | .008 | **5.125** | .043 | .508 | 1.003 | .082 | .555 |
|  | DF | - | 142.484 | **42.692** | 16.838 | 20.014 | 37.431 | 22.620 | 327.643 |
| RFS1 | P | - | .712 | .177 | .500 | .042 | .681 | .653 | .280 |
|  | F | - | .137 | 1.876 | .470 | 4.506 | .171 | .206 | 1.264 |
|  | DF | - | 148.168 | 46.032 | 22.898 | 30.763 | 38.335 | 31.103 | 13.894 |
| Distance moved | P | - | .133 | **.003** | .084 | .398 | .101 | .970 | .398 |
|  | F | - | 2.288 | **9.430** | 3.146 | .730 | 2.773 | .001 | .753 |
|  | DF | - | 150.198 | **68.729** | 36.876 | 42.154 | 66.067 | 38.904 | 16.947 |
| K | P | .865 | .075 | **-** | **-** | **-** | .458 | **-** | .944 |
|  | F | .029 | 3.211 | **-** | **-** | **-** | .563 | **-** | .006 |
|  | DF | 256.676 | 141.121 | **-** | **-** | **-** | 36.108 | **-** | 3.875 |
| Total length | P | .972 | .373 | **-** | **-** | **-** | **<.001** | **-** | **<.001** |
|  | F | .001 | .794 | **-** | **-** | **-** | **40.287** | **-** | **55.238** |
|  | DF | 265929245.871 | 27796.882 | **-** | **-** | **-** | **8661.908** | **-** | **4488.734** |
| Mass | P | .075 | **.003** | **-** | **-** | **-** | **<.001** | **-** | .082 |
|  | F | 3.204 | **8.915** | **-** | **-** | **-** | **19.061** | **-** | 3.138 |
|  | DF | 266.056 | **115.989** | **-** | **-** | **-** | **172.936** | **-** | 59.426 |
| SGR | P | **-** | **<.001** | **-** | **-** | **-** | **<.001** | **-** | .095 |
|  | F | **-** | **13.719** | **-** | **-** | **-** | **20.666** | **-** | 3.739 |
|  | DF | **-** | **118.238** | **-** | **-** | **-** | **31.723** | **-** | 6.938 |
| dpi | | 0 | 30 | 60 | 90 | 150 | 270 | 330 | 360 |
